# Efficiency vs Depth in AI-Generated and Human-Synthesized Thematic Analyses of Ugandan Women’s Experiences of Obstetric Fistula: Comparative Qualitative Study

**DOI:** 10.2196/94809

**Published:** 2026-10-09

**Authors:** Madeline Pechilis, Monica Getahun, Hadija Nalubwama, Patrick Eyul, Florence Ebem, Alison M El Ayadi

**Affiliations:** 1 Institute for Global Health Sciences University of California, San Francisco San Francisco, CA United States; 2 Department of Obstetrics and Gynecology College of Health Sciences Makerere University Kampala, Central Region Uganda; 3 Infectious Diseases Research Collaboration Kampala, Central Region Uganda; 4 School of Medicine University of California, San Francisco San Francisco, CA United States; 5 Department of Obstetrics, Gynecology and Reproductive Sciences University of California, San Francisco San Francisco, CA United States

**Keywords:** artificial intelligence, ChatGPT, data analysis, fistula, generative AI, interviews as topic, large language models, obstetric surgery, qualitative research, Uganda

## Abstract

**Background:**

Limited recent literature has evaluated the use of large language models (LLMs) in the qualitative analysis of health data; more research is needed to expand the generalizability of LLM use and to evaluate potential ethical considerations.

**Objective:**

Our research sought to (1) describe the process of using AI to analyze qualitative in-depth interview data, (2) identify similarities and differences between the human and AI-generated analyses to compare the quality and rigor of the two techniques and describe the strengths and weaknesses of each approach, and (3) make recommendations regarding the bounds of ethics and the role of researcher bias in AI-assisted qualitative research.

**Methods:**

Nested within a larger mixed methods study, 17 Ugandan women recovering from female genital fistula surgery participated in hour-long semistructured interviews exploring their mental, physical, and overall health trajectories. Each interview lasted about an hour and was audio-recorded. Following translation and transcription, the data underwent thematic analysis by humans and by Versa, a University of California, San Francisco (UCSF) developed LLM powered by ChatGPT-4o. The AI analysis was conducted using 2 strategies: inductively and deductively. Finally, the analytic outputs were compared by MP and reviewed by AME and MG to evaluate the code frequency and alignment (most frequently used codes and equivalent concepts vs unique concepts between human and AI codes), thematic robustness (differentiation of distinct concepts and labels that convey substantive findings), analysis quality (narrative depth and excerpt accuracy), and efficiency of each method (total person-hours spent on comparable analytic tasks).

**Results:**

A comparative analysis revealed significant thematic overlap between the human-synthesized and AI-generated outputs, though notable differences in granularity and efficiency emerged. When inductively coding, Versa identified 39 codes, whereas human researchers used a more expansive set of 54 codes. While Versa’s thematic analyses were generally accurate regarding how the interview data were represented in the analyses, the human-synthesized analysis was more robust because it incorporated compelling excerpts, and the summary content provided greater depth and range that the Versa analyses lacked. The disparity in efficiency was stark: the human analysis took approximately 15 hours to complete, while Versa produced the analysis in about 2.5 hours.

**Conclusions:**

While Versa significantly expedited the initial coding phase, it still relied heavily on human researchers to create appropriate prompts and input all the data into the chat. Versa’s inability to replicate the narrative depth and description of human synthesis suggests that LLMs currently lack the interpretive sensitivity required to capture the lived experiences. Using Versa alone does not currently yield a high-quality analysis; significant human engagement is needed to maintain ethical and interpretive rigor.

**Trial Registration:**

ClinicalTrials.gov NCT05437939; https://clinicaltrials.gov/study/NCT05437939

## Introduction

### Use of AI in Qualitative Health Research

The use of AI in qualitative research, though nascent, is rapidly evolving. Incorporating AI into health data analysis is a promising tool to reduce the burden on researchers and increase efficiency [1]. AI in academic research is relatively new; past methods have relied heavily on human interpretation and analysis to navigate the data, interpret themes, and draw attention to important findings. The validity, ethics, and documentation of the use of AI in replacing humans in qualitative research are currently unknown but hold promise.

Limited recent literature has provided an initial evaluation of the use of ChatGPT in qualitative health data analysis [1-5]. ChatGPT is a popular AI large language model (LLM) with capabilities that include assisting with tasks such as writing or coding and providing explanations and definitions. Some institutions have developed their own LLMs that use ChatGPT algorithms, such as Versa, which is an LLM created by the University of California, San Francisco (UCSF). Increasing efficiency by reducing the time spent coding transcripts and developing themes is a substantial potential benefit that LLMs could provide [1,6,7]. In the limited comparative literature, human coding and analysis have been found to take 3.5 to 6 times longer than AI-assisted coding and analysis [6,7]. AI qualitative analysis is also effective at developing themes from transcripts, but early research indicates the quality of the output is not robust, raising significant issues with reliability if used as the sole source of analysis [1-3,5-7]. Studies suggest that LLMs can be used to independently code the data to validate the initial human coding, and may identify codes in the data that human researchers missed [1,2].

ChatGPT successfully identifies broad themes but is less consistent at producing valuable subthemes and specific codes [1,3]. Another concern is that researchers may blindly accept the LLM findings and fail to critically evaluate the output [4]. Although previous studies recommended that coding and identifying themes should be the extent of AI use in qualitative data analysis, this still suggests that AI-assisted qualitative analysis may enable more expansive and comprehensive research through the combined efforts of the human researcher and LLMs [1,5].

### Use of AI in Maternal Morbidity Qualitative Research and Beyond

An important evidence gap exists in understanding the use of LLMs for qualitative analysis of women’s experiences of maternal morbidity in sub-Saharan Africa, given the importance of cultural competence in such analysis. This limitation is particularly relevant for applying LLMs to our topic of obstetric fistula due to the complex impacts of the condition and the unique characteristics of this study population. ChatGPT was created by a San Francisco–based AI research company that exists in cultural contexts that are very different from the context of our study. ChatGPT is oriented toward American culture and Western values, and does not capture other cultural values, such as religion, parent-child ties, economic security, physical security, and gender equality, whether prompted in English or other languages [8-10]. Other cultural dimensions that may introduce bias in ChatGPT include individualism/collectivism, independent/interdependent self-construal, and relationship mobility [11]. This cultural bias may occur from ChatGPT inadvertently overrepresenting the cultural narratives of its training data, which is predominantly in English [10-12]. Conversely, ChatGPT is vulnerable to developing oversimplified views of cultural groups that it has limited information sources on [11]. Women in our study have low health literacy and educational attainment, and speak local languages, which may compromise the performance of AI analysis. With these unique characteristics, AI may not be capable of comprehensive interpretation of these types of data.

AI analysis via LLMs is a promising methodological element that is relatively new to the qualitative research field and has the potential to both benefit from and inform qualitative studies across disciplines. In this study, we conducted human and AI qualitative analyses of the same data to assess the comparability of their content and techniques. This analysis expands upon the limited research on qualitative LLM analysis, and the unique cultural contexts of this study population may provide insight into the generalizability of AI-generated qualitative analysis. Furthermore, the unique characteristics of the study population provide an opportunity to examine how an LLM successfully or unsuccessfully navigates cultural nuances in the data and manages bias. Our research aims to describe the process of using AI to analyze qualitative in-depth interview (IDI) data, identify the similarities and differences between the human and AI-generated analyses, and make recommendations regarding the ethics and the role of researcher bias in AI-assisted qualitative research.

## Methods

### Study Design Overview

Data used in this secondary qualitative analysis are from an ongoing mixed methods study, *Identifying Opportunities for Prevention of Adverse Outcomes Following Female Genital Fistula Repair* (NCT05437939), which combines a longitudinal cohort study with IDIs [13]. Each year, fistula affects roughly 450,000 women, with the highest prevalence in sub-Saharan Africa, and commonly causes urine and fecal incontinence, vaginal pain, and impaired mobility [14-18]. Study participants were recruited from 8 fistula repair sites across Uganda (Figure 1); a nested purposive sample of participants was invited to complete IDIs. This analysis used interviews from 17 women who enrolled in the cohort study and were subsequently interviewed.

**Figure 1:**
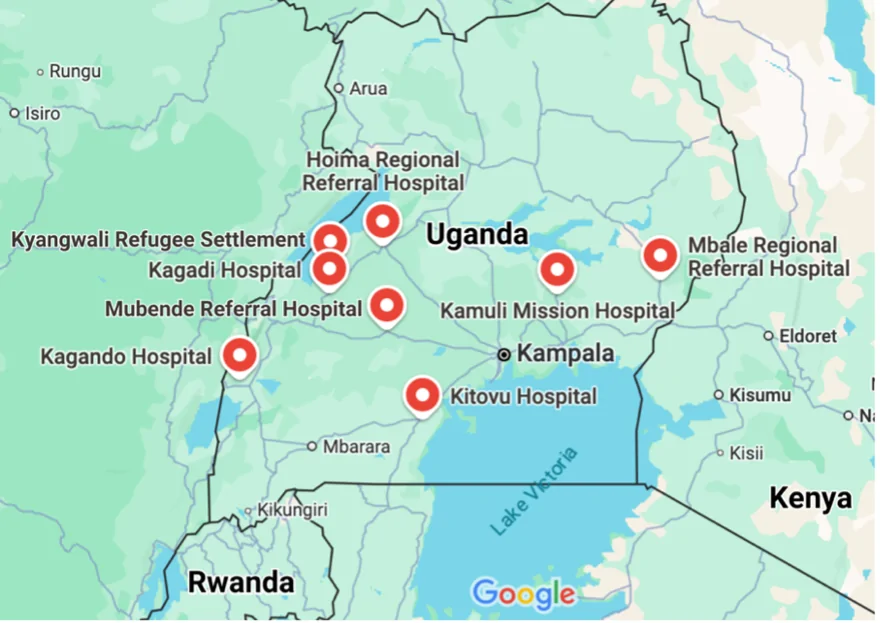
Partnering Ugandan fistula repair sites where women with fistula were treated and recruited for in-depth interviews (n=8).

### Participant Recruitment and Enrollment

Detailed study procedures are published elsewhere [13]. Briefly, longitudinal cohort participants had vesicovaginal fistula and underwent fistula repair at study sites, which resulted in confirmed fistula closure per clinical guidelines [13]. Individuals unable to participate in cohort follow-up activities were excluded. Research assistants screened and recruited participants at least 24 hours after the fistula repair surgery was performed, engaging them in a robust informed consent process.

### Qualitative Subsample Selection

Women for qualitative IDIs were purposively selected from the parent study cohort to ensure variability in regional and postrepair recovery experience [13]. Potential participants were recruited via phone or in-person; those who expressed interest provided separate, written informed consent prior to data collection [13].

### Data Collection

A semistructured, open-ended IDI guide was developed to evaluate women’s fistula-related experiences across several domains: surgical repair, food security, social support, intimate partner violence, and access to post–fistula-repair care, to inform intervention opportunities to prevent adverse health outcomes after fistula repair surgery (Multimedia Appendix 1 [13]). To ensure cultural and linguistic nuance, an experienced qualitative researcher conducted the IDIs in a private setting using local languages, including Runyankore, Luganda, Runyoro, Rutooro, and Kinyarwanda [13]. Each IDI lasted approximately an hour [13]. Sociodemographic characteristics of the participants (age, marital status, education, employment, and monthly household income) were collected within the parent study and linked with the IDI participants for analysis. For the current analysis, 17 audio-recorded IDIs were translated into English and transcribed [13].

### Data Analysis

#### Human-Synthesized Analysis

Following data collection, researchers used a multistage process to disassemble and reassemble the data into meaningful themes, aiming to identify patterns of phenomena across and within individuals [19,20]. Data were coded using a hybrid deductive and inductive approach. The deductive codes target the objectives of the parent study, to understand facilitators and barriers influencing implementation of supportive postrepair behaviors and multilevel influences on postrepair recovery, and were derived from the IDI guide and established theory: the Capability, Opportunity, Motivation–Behavior (COM-B) system and socioecological model [21,22]. Inductive codes emerged iteratively to capture unexpected participant insights. The diverse research team consisted of 4 US-based and 2 Uganda-based researchers with qualitative research experience and backgrounds spanning maternal health, global health, sub-Saharan Africa, and medicine. One team member holds a Doctor of Science, 1 team member holds a Master of Public Health, 3 team members hold a Master of Science, and 1 team member holds a Bachelor of Science.

To ensure intercoder reliability, the 6 team members (led by MG under the supervision of AME) developed the codebook through 3 iterative rounds of application and revision. Each round consisted of each team member blind coding the same transcript and then meeting to review code applications. This reflexive process allowed the team to acknowledge their own positionality and potential biases in interpreting sensitive data. The first 2 rounds used the same transcript, which was selected because it was long and detail-heavy, and the third round used a new transcript to ensure the codebook was comprehensive enough for a variety of interview data. Discrepancies were resolved through group consensus until a finalized codebook was established (Multimedia Appendix 2). Coding (completed by MP, HN, PE, and FE) was facilitated by Dedoose, a cloud-based qualitative analysis software program for text labeling, with a second coder assigned to each transcript to verify code application [23]. All 4 team members coded the 2 transcripts used to develop the codebook, and 1 team member (MP) cleaned up the code applications after the codebook was finalized. The remaining 15 transcripts were divided up among team members: MP was the first coder for 5 transcripts and the second coder for 4 transcripts, HN was the first coder for 4 transcripts and the second coder for 5 transcripts, PE was the first coder for 3 transcripts and the second coder for 3 transcripts, and FE was the first coder for 3 transcripts and the second coder for 3 transcripts. The first coder and second coder assignments varied, so the same 2 team members were not consistently working on the same transcripts. Finally, the team synthesized these codes into overarching themes through thematic mapping, collaboratively identifying themes and their relationships to specific codes, ensuring the resulting analysis reflected women’s lived experiences [19,20,24].

#### AI-Generated Analysis

Following human analysis, the previously deidentified transcripts were processed using Versa, a UCSF-developed LLM. Versa is a private and secure generative AI platform using OpenAI’s GPT-4o model and was selected due to its enhanced security protocols; unlike public generative AI platforms, it does not retain input data for model training, thereby ensuring Health Insurance Portability and Accountability Act (HIPAA) compliance and participant confidentiality [25,26]. One team member (MP) conducted all of the AI analyses for consistency.

To replicate the rigor of human-driven qualitative research, we developed standardized prompts. First, Versa was questioned about uploading documents and exporting chat contents. Initial feasibility testing revealed that Versa could not accept batch file uploads, requiring transcripts to be input sequentially via text. Next, Versa was asked about performing a qualitative analysis of interview data, and initial prompts were tested and went through four rounds of revision to optimize the output’s format alignment with the human qualitative analysis to facilitate comparability. Revisions consisted of expanding upon the research question to obtain a more detailed analysis, requesting excerpts in the analysis, attempting to condense the prompt, and the final revision included enough instruction for Versa to produce an analysis with comparable layout and level of detail. To mitigate “hallucinations” or thematic drift, we used a new chat session for each transcript. We conducted 2 distinct analytical passes: Versa Analysis 1 used an inductive approach, instructing the model to generate codes and themes independently (Figure 2); Versa Analysis 2 used a deductive approach, providing the model with the validated human codebook (Multimedia Appendix 2) and instructing it to generate themes (Figure 3). To prevent intrachat bias or memory lag, each of the 17 transcripts was analyzed in a stand-alone chat session using the finalized prompts (Multimedia Appendices 3 and 4). Because the Versa interface does not archive chat session history, all outputs were manually exported into a Word document for comparative analysis.

**Figure 2:**
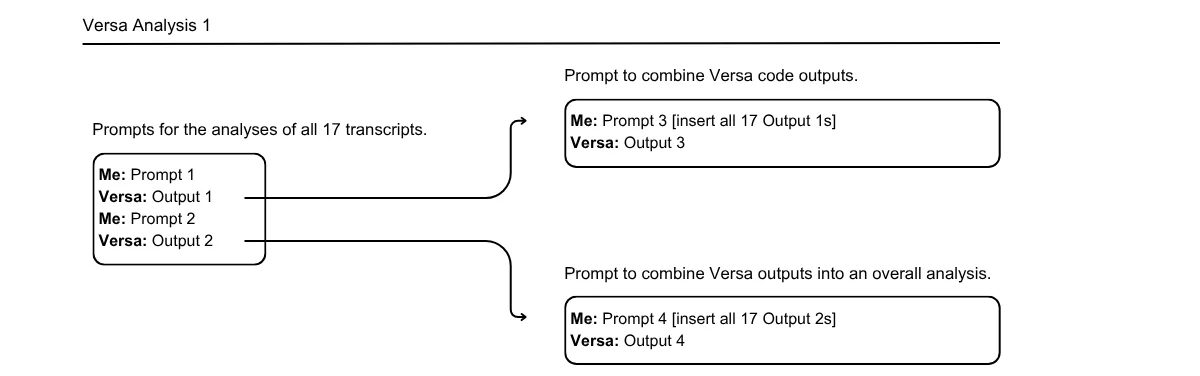
Flowchart of Versa Analysis 1 prompts and combining outputs into an overall qualitative analysis (Multimedia Appendix 3). Each bubble represents a new chat session.

**Figure 3:**
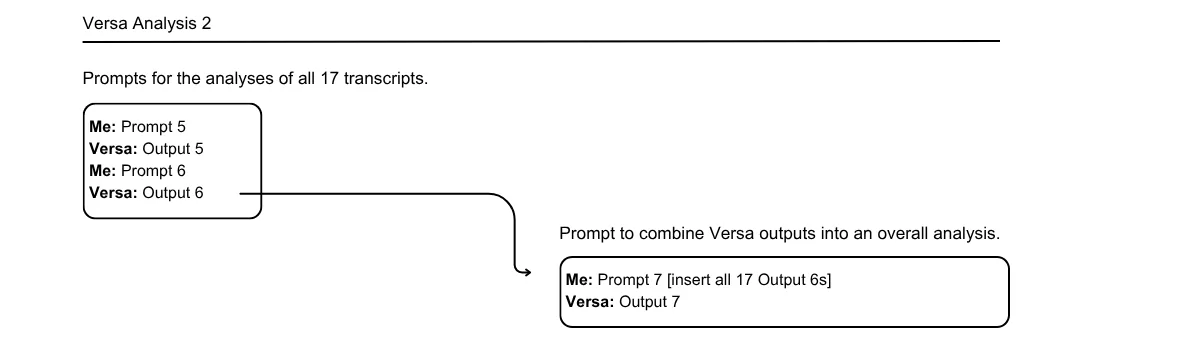
Flowchart of Versa Analysis 2 prompts and combining outputs into an overall qualitative analysis (Multimedia Appendix 4). Each bubble represents a new chat session.

Versa Analysis 1 output was broken down into 2 elements: identification of codes and themes, and an overall analysis of the transcript. Versa replied to prompt 1, “I am going to give you interview transcripts, and I want you to qualitatively analyze them. Identify codes and provide excerpts from the transcript as examples. Identify themes and provide excerpts from the transcript as examples...” (Multimedia Appendix 3) with codes and themes. Versa replied to prompt 2, “Now, create an overall analysis of the transcript. The research question is: What are Ugandan women’s experiences of obstetric fistula, fistula repair surgery, and recovery...” (Multimedia Appendix 3) with an overall analysis. Prompts 1 and 2 were delivered in the same chat session. The entirety of the Versa output for both prompts was copied and pasted into a single document and labeled by study ID. By the end, there were 17 analysis documents, one for each transcript.

The next step of Versa Analysis 1 consisted of combining the 17 Versa outputs to the codes prompt into one document and delivering prompt 3, “I am going to give you a qualitative analysis of codes and excerpts for 17 different study IDs. I want you to condense all of the 17 analyses into one final analysis of codes and excerpts” (Multimedia Appendix 3). And combining the 17 Versa outputs to the overall analysis prompt into a second document and delivering prompt 4, “I am going to give you an overall qualitative analysis for 17 different study IDs. I want you to condense all of the 17 analyses into one final analysis” (Multimedia Appendix 3). Prompts 3 and 4 were delivered in separate chat sessions. Output 3 did not preserve the code frequency through the condensation of Output 1, so code frequency and alignment were reconstructed separately. Frequency was determined by the number of times Versa used a given code in all 17 Output 1s. The total Versa code count was determined by combining all generated codes and removing duplicates. Output 4 Overall Analysis is the Versa Analysis 1 material used in the thematic comparison to the human analysis.

Versa Analysis 2 was prompted similarly. Prompt 5 instructed, “I am going to give you interview transcripts, and I want you to qualitatively analyze them using the uploaded codebook. Identify themes and provide excerpts from the transcript as examples...” (Multimedia Appendix 4). In the same chat session, prompt 6 instructed, “Now, create an overall analysis of the transcript. The research question is: What are Ugandan women’s experiences of obstetric fistula, fistula repair surgery, and recovery...” (Multimedia Appendix 4). There were 17 analysis documents, one for each transcript, at the end of this stage. As with Versa Analysis 1, the following step of Versa Analysis 2 was to combine the 17 Versa outputs into one document and instruct Versa to combine the outputs for an overall analysis, as shown in prompt 7, “I am going to give you an overall qualitative analysis for 17 different study IDs. I want you to condense all of the 17 analyses into one final analysis” (Multimedia Appendix 4). Decision parameters for the condensation of 17 Versa outputs into one final analysis were intentionally left to the LLM. The output to this prompt, Output 7 Overall Analysis, is the Versa Analysis 2 material used in the comparison to the human analysis.

#### Human Analysis and AI Analysis Comparison

##### Overview

Following the completion of both analytical arms (Multimedia Appendices 5 and 6 [27]), 1 team member conducted and 2 team members independently reviewed a systematic comparison across four domains: code frequency and alignment, thematic robustness, analysis quality, and efficiency. The 2 reviewers (AME and MG) agreed with all comparison judgments made by the initial team member (MP) and noted elements of the results that needed to be expanded upon for additional clarity and depth. The initial team member addressed all reviewer notes. The human analysis, which had been finalized through iterative coding and consensus meetings prior to any AI analysis, served as the gold standard for these evaluations. The operationalized comparison criteria are defined in italics in the following descriptions.

##### Code Frequency and Alignment

We first compared the inductive codes from Versa Analysis 1 against the human baseline to identify overlaps and unique outliers. *Code frequency (human analysis)* was measured by the number of times the code was applied to excerpts in all 17 transcripts. *Code frequency (Versa)* was measured by how many transcripts used the given code. *Code alignment* was defined as equivalent concepts represented vs unique concepts, with conceptual correspondence rather than identical wording used to categorize the same concepts as equivalent codes. Both code frequency and alignment were criteria specified before analysis.

##### Thematic Robustness

We evaluated the overarching themes from both Versa Analysis 1 (inductive) and Versa Analysis 2 (deductive) against the human analysis themes. To manage the data volume, this comparison focused specifically on the physical and emotional dimensions of the fistula experience. *Thematic robustness* was defined by the specificity of themes, differentiation of distinct concepts and experiences, coverage of salient concepts represented in the analysis, and whether theme labels conveyed substantive findings rather than restating the interview domain. These were prespecified criteria that expanded postanalysis to include comparison of theme labels.

##### Analysis Quality

We assessed the narrative depth of the summaries and the accuracy of the excerpt selections, comparing Versa Analysis 1 (inductive) and Versa Analysis 2 (deductive) against the human analysis. *Narrative depth* was defined by the contextualization of participant experience, linkage between experiences/conditions and their consequences, preservation of complexity and nuance within an account, and integration of excerpts into an interpretative narrative rather than summarizing topics. *Excerpt accuracy* was defined by transcript verbatim fidelity, correct participant attribution, sufficient context to make the excerpt meaning interpretable, inclusion of substantively important material, and relevance to the theme. Narrative depth was a prespecified criterion, while excerpt accuracy was not.

##### Efficiency

We recorded the total person-hours required for the manual analysis vs the cumulative time for AI prompting and output management. *Efficiency* was defined as person-hours for comparable analytic tasks, with non-comparable Dedoose coding time explicitly separated. This was a prespecified criterion.

Researchers maintained a structured research journal to capture the reflexive element of using AI during the analytical process. These memos documented the “prompt engineering” required to guide the LLM, identified nuances lost during automation, and tracked the evolving relationship between the researchers and the LLM.

##### Excerpt Comparison Example—Applying Criteria of Narrative Depth

The following two excerpts are from emotion-related themes in the human analysis and Versa Analysis 2 (deductive).

###### Human Analysis

“A 44-year-old woman said that there are times she doesn’t go to work if she doesn’t have diapers due to a lack of money, because when she uses cloths to pad herself, the odor is too strong even immediately after washing. She described the emotional toll of needing padding to cope with fistula urine incontinence: *‘There’s no [feeling] of happiness at all*... *my heart is not at peace. I am not happy with myself. I keep asking myself why the urine keeps leaking. Sometimes you go to dry the cloths on the hanging line. People keep seeing one soiled towel and cloth. Every single time. When new neighbors come, you get worried. When you see people who know you that you keep drying the same thing. When you have no soap, you just dry them without washing. Whoever passes smells them. So, you feel your heart is tired.’’*”

###### Versa Analysis 2 (Deductive)

"The emotional toll of fistula was profound. Women described feelings of shame, despair, isolation, and loneliness. Many were stigmatized and ridiculed by their communities, facing rejection at work, in social settings, and even within their families. Spousal relationships often deteriorated due to the condition, leading to separation or abandonment.

Excerpts: [...]

-
  ‘When new neighbors come, you get worried. When you see people who know you...Whoever passes smells them. So, you feel your heart is tired.’ (Participant 8)"
27

###### Narrative Depth Criteria Applied to the Human Analysis

Contextualization of participant experience—yes, the excerpt explains the participant relies on cloths to pad herself when she does not have money for diapers, which provides context for the quote about cloths. Linkage between experiences/conditions and their consequences—yes, the excerpt connects having fistula urine incontinence to using cloths for padding, and the emotions related to both experiencing leakage and relying on cloths, and how that connects to strained interpersonal relationships with neighbors. Preservation of complexity and nuance within an account—yes, the excerpt includes the full quote to detail how lack of money impairs the participant’s ability to manage urine incontinence and the resulting negative emotions. Integration of excerpts into an interpretative narrative rather than summarizing topics—yes, the entire paragraph is dedicated to capturing this participant’s experience rather than focusing exclusively on the emotions.

###### Narrative Depth Criteria Applied to the Versa Analysis 2 (Deductive)

Contextualization of participant experience—yes and no, the quote identifies experiencing worry regarding neighbors, which connects to the first paragraph stating participants were stigmatized by neighbors, but too much of the quote is excluded to understand what the participant is referring to and why she is worried. Linkage between experiences/conditions and their consequences—no, it is unclear how the quote relates to fistula. Preservation of complexity and nuance within an account—no, too many details were omitted from the quote to explain the participant’s experience. Integration of excerpts into an interpretative narrative rather than summarizing topics—no, the quote is provided as a bullet point after a short paragraph summarizing the theme.

#### Ethical Considerations

The use of LLMs in health research necessitates rigorous safeguards to maintain participant confidentiality. To mitigate data privacy risks, all transcripts were deidentified before the human and AI analyses. Participants received 40,000 Ugandan Shillings (~US $11) as an appreciation of their time. Participant incentive was the standard amount used in this research setting and was not determined relative to participants’ monthly household income. Study procedures were reviewed and approved by the UCSF Human Research Protection Program, Committee on Human Research (IRB# 21-33559), Makerere University School of Medicine Research and Ethics Committee (reference number 2021-277), and the Uganda National Council for Science and Technology (reference number HS2033ES) [13]. While the original informed consent process did not explicitly mention AI or LLMs, it did stipulate that deidentified data could be used for secondary analyses without further permission. Because all data were deidentified and processed through a secure LLM without data retention, Versa, the current analysis remained within the established bounds of participant consent and HIPAA regulations [13].

## Results

### Overview

The demographic characteristics of the 17 study participants, collected at the baseline visit of the parent study, are summarized in Table 1. Participants ranged in age from 18 to 49 years. The majority of the cohort (n=8) reported “some primary school” as their highest level of education. Most women were in a marriage or domestic partnership (n=9) and were unemployed (n=8). Socioeconomic vulnerability was further reflected in the self-reported household income, which ranged from UGX 0 to UGX 800,000 (~US $223) per month, with a median monthly income of UGX 100,000 (~US $28).

**Table 1:** Baseline sociodemographic characteristics of Ugandan women with fistula (N=17).

Characteristic			Value, n
**Age (years)**			
	18-19	5	
	20-29	3	
	30-39	4	
	40-49	5	
**Marital status**			
	Single, never married	3	
	Married/partnership	9	
	Separated	3	
	Widowed/divorced	2	
**Education**			
	None	1	
	Some primary	8	
	Completed primary	1	
	Some secondary	4	
	Completed secondary or higher	3	
**Employment**			
	Not employed	8	
	Housewife	5	
	Informal employment for wages	3	
	Self-employed	1	
**Monthly household income (UGX 10,000=US $2.8)**			
	0	2	
	1-5	5	
	10	2	
	20	4	
	30+	4	

### Comparability of Human-Synthesized and AI-Generated Analyses

Across the full analysis, human researchers developed a total of 54 initial codes (19 parent codes and 35 child codes), and Versa Analysis 1 (inductive) identified a total of 39 initial codes. Versa independently generated codes for each transcript and only created child codes for 6 of the 17 transcripts. Of the 39 codes used by Versa, 4 of them were not used by human researchers: *postsurgery recovery process*, *multiple surgeries*, *fistula and surgery history*, and *understanding guidance*. Human researchers identified more specific codes, such as *postrepair symptoms and experiences*, *health information access*, and the *impact of counseling and implementation on healing*, to break down different elements of the recovery process rather than group them together like Versa. The *multiple surgeries* code used by Versa encapsulates unsuccessful surgery, repeated surgery, first surgery, second surgery, number of surgeries, and timeline of surgeries. These distinctions between surgeries were not included in the human codebook because, in the transcripts, the participants did not always specify which exact surgical experience they were describing if there were multiple. Similarly, only some transcripts provided specific details on the participant’s fistula and surgical history because the parent study mainly relied on the quantitative methods to obtain that data. The Versa code *understanding guidance* was only used for 2 transcripts, which is likely why the human researchers did not include it in their codebook. The human researchers had adjacent codes for any description of health information they received and for the impact of the guidance, which were far more common across all the transcripts. On the other hand, 19 codes selected by human researchers were not used by Versa, which are indicated by an asterisk in the codebook (Multimedia Appendix 2). Between the human researchers and Versa, there were 35 equivalent codes.

The 3 initial codes most commonly applied by human researchers are *providers/health care workers*, *informational support*, and *implementing postsurgical guidelines* (Figure 4). The 3 initial codes most commonly applied by Versa Analysis 1 (inductive) are *health information access*, *surgical experiences*, and *postrepair symptoms and experiences* (Figure 4). There are 4 codes, designated by arrows in Figure 4, that were in the top 10 most commonly applied by both human researchers and Versa: *postrepair symptoms and experiences*, *intimacy and sexuality*, *info or support from relatives/family members*, and *physical function and activities*. The code counts on the x-axis are not shown because, for the human analysis, the code count was how many times the code was applied to excerpts in all 17 transcripts, whereas for Versa, the code count was determined by how many transcripts Versa generated the given code. The code titles for *info or support from relatives/family members* and *implementing postsurgical guidelines* were slightly altered for clarity (Multimedia Appendix 2 for original code titles).

**Figure 4:**
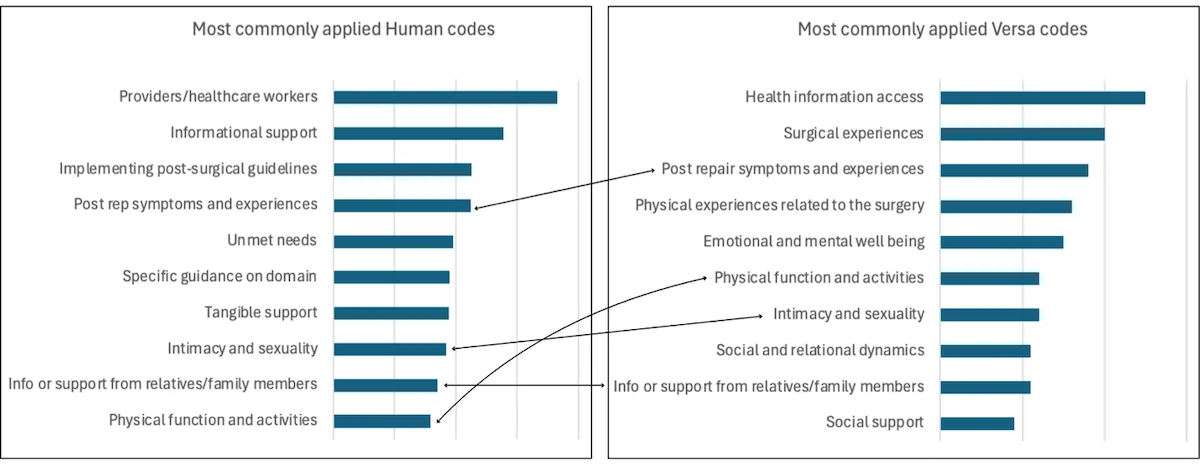
Most commonly applied initial codes in human and Versa qualitative data analyses.

The themes included in the human analysis were more descriptive than the Versa analysis. For the experiences of fistula part of the analysis, the Versa Analysis 1 (inductive) themes were “physical symptoms” and “emotional impact,” which do not provide any insight into key concepts that are not already available from the research question. Similarly, Versa Analysis 2 (deductive), which only generated themes and used the human codebook, used the themes “physical symptoms and impact” and “emotional distress,” but unlike Versa Analysis 1, it also included “coping mechanisms” as a theme. Comparatively, the human analysis themes of “urinary incontinence while sleeping,” “wearing a diaper or padding,” “negative emotions,” and “self-hatred” are more informative about the findings of the analysis.

The quality of the Versa excerpts was notably weaker than those selected for presentation in the human analysis. Versa Analysis 1 contained 4 excerpts in quotation marks for each theme, but many excerpts for each theme were misrepresented as direct quotes. Versa presented them within quotation marks, but the quoted content is nonexistent in the respective transcript. Half of the excerpts provided by Versa regarding experiences were misrepresented as direct quotes. This was especially obvious when a Versa quote referred to the participant in third person. For example, this part of the Versa output for the emotional impacts of fistula is misrepresented as a direct quote:

“Emotionally, the respondent faced significant distress and embarrassment due to the fistula.” (Participant 4)

27

Versa Analysis 2 also struggled to ensure that all the excerpts were high-quality and impactful. Two of the Versa Analysis 2 excerpts were paraphrases, not direct quotes, and one of the excerpts is attributed to the wrong participant. There were also missed opportunities in both Versa analyses to include a variety of participants’ voices: of the 8 excerpts in Versa Analysis 1, only 5 participants were represented, and of the 11 excerpts in Versa Analysis 2, only 8 participants were represented.

Furthermore, several of the excerpts from Versa Analysis 2 lacked enough context to maximize their impact. For example, the quote below describes the emotional toll of stigmatization. The participant washes cloths soiled from incontinence every day, but on days when she cannot afford soap, she just hangs them to dry and worries about the smell.

“When new neighbors come, you get worried. When you see people who know you...Whoever passes smells them. So, you feel your heart is tired.” (Participant 8)

27

Without the information provided in the lead-in sentence to the excerpt, written by human researchers, it is unclear from Versa’s quote selection *what* smells, *why* it smells, and how it relates to fistula. Additionally, one of the excerpts from Versa Analysis 2 is one sentence too short and omits a very powerful emotional statement. The excerpt is, “Some people used to come and tell me that you won’t heal. They can’t operate a bladder. It is just a polyethene bag. Can you pass a needle through a polyethene bag? You are meant to die.” However, the next sentence in the transcript is, “So, I said to myself, why don’t I commit suicide and die?” Suicidal thoughts are an extreme and notable emotional toll of fistula, and the Versa excerpt failed to include that detail from the transcript.

The inclusion or exclusion of culturally relevant vocabulary was also considered when comparing the quality of analyses. The LLM content did not maintain the use of words and phrases that were uncommon in United States English but appeared in the transcripts after being translated to English from Ugandan languages. For example, in the interviews, many participants referenced “digging” and carrying a “jerrycan” when recalling their day-to-day activities. The human analysis maintained the use of the unique vocabulary by capturing it in quotes, but “digging” and “jerrycan” were eliminated in the LLM analysis. However, the key concept of physical activity, to which this unique vocabulary was associated, was still included in the LLM analysis.

A considerable difference between the human-synthesized and the AI-generated analyses was the amount of time spent on each analysis. The total time spent on human analysis was about 74 hours: about 9 hours developing and revising the codebook, about 59 hours applying the codes to the transcripts, and about 6 hours writing the analysis. Identifying codes and themes and producing a written analysis took human researchers about 15 hours (9 hours on the codebook and 6 hours on the analysis); contrastingly, the Versa Analysis 1 (inductive) process of generating codes and an analysis only took about 2.5 hours. The bulk of the human time spent during the analysis process was on applying codes to the transcripts in Dedoose, which took about 59 hours. Versa identified codes but did not apply the codes to the transcripts like the human researchers did in Dedoose because that is beyond what Versa is capable of.

## Discussion

### Principal Findings

This analysis of physical and emotional experiences with fistula using transcripts describing Ugandan women’s experiences living with and recovering from fistula had similar findings to previous research: the human-synthesized analysis and AI-generated analysis (Versa Analysis 1) produced similar codes but diverged in the quality of themes and excerpts [1-3,5-7]. Both of the Versa analyses had informative thematic summaries, but without the inclusion of adequate excerpts, the outputs severely lacked the narrative-style analyses that are typical of qualitative research to organize the thematic interpretations and share participants’ experiences. The human analysis maintained nuanced participant stories, while the LLM analyses only provided an impersonal summary of them. Almost 9 hours of human analysis were spent on team meetings to revise the codebook and on updating the codebook in Dedoose to reflect the changes after every meeting. Since the LLM-produced codes (Versa Analysis 1) were very similar to the human-developed codes, the creation of the codebook may represent a reliable use of LLMs within the qualitative analysis process [1,2,5]. Similar to the ratio of a previous study on AI-assisted coding and analysis, Versa was 6 times faster than the human researchers (2.5 vs 15) [7]. The results of this study supported and expanded upon the findings of previous literature on the use of LLMs in qualitative analysis, concluding that the LLM successfully identified codes for the qualitative data, but the thematic analysis was not as robust as human analysis [1-3,5-7].

This research provided a new perspective on the use of qualitative LLM analysis of data from a population with a different cultural background from that of the data ChatGPT was trained on. The maternal health data collected in Uganda, a low- and middle-income country (LMIC) in sub-Saharan Africa, highlighted a gap in the cultural competence of LLM qualitative analysis [28]. Even though the transcripts were translated from local Ugandan languages to English, culturally relevant vocabulary was not maintained in the AI analyses, which suggests an LLM may overrepresent language it is more familiar with from their training material [8,10,11]. LLMs trained predominantly with Western data, culture, and values are not yet a reliable source of qualitative analysis for LMIC research contexts [12]. In future research using an LLM to analyze data from culturally diverse populations, there is a demonstrated need for ChatGPT to improve cultural alignment and avoid consequences of cultural dominance such as misinterpretations, increasing inequality, and the loss of cultural diversity [9,10,12]. There should be an ethical consideration for the preservation of culturally relevant vocabulary and terminology, which also addresses a potential source of LLM bias. To accomplish this, LLMs may require more comprehensive training datasets and instruction on how to include concepts that may be unfamiliar to them, rather than overlooking them [11]. This research explored language and terminology as a source of LLM bias, but there may be a multitude of other sources of bias that need to be examined and addressed before LLMs consistently produce reliable qualitative analyses of LMIC data.

### Strengths and Limitations

A diverse team of researchers in Uganda and the United States worked collaboratively to conduct the human qualitative analysis of the interview data, and 2 team members from Uganda and 2 team members from the United States worked closely together to double-code the transcripts and review findings. This ensured that the cultural components of the interview data were not lost during translation or misunderstood by the team members in the United States. During the coding process, the 4 designated team members worked independently to code the same 2 transcripts, which informed the development of the codebook and ensured intercoder reliability. For the remainder of the transcripts, 1 team member was assigned to be the primary coder, and a second team member was assigned to be a secondary coder and thoroughly review the work of the first coder.

The sample size was small (N=17), which may limit the generalizability of the results. Generalizability would be improved with more participants, and if the data could be analyzed within the same AI prompt. ChatGPT models are released and/or updated every few months, so the capabilities of the ChatGPT-4o-based model used in this study may differ from those of the most recent ChatGPT models [29]. Versa’s output consistency across sessions was not evaluated. A critical feature the Versa LLM lacked was the ability for the human researchers to upload the interview transcripts at the same time. Relying on copying and pasting the transcripts into the chat box increased the total time spent on the AI-generated analysis, and it limited the ability of Versa to analyze all the transcripts simultaneously. The synthesis step of combining 17 outputs into one analysis is itself an opaque LLM operation, so information may have been lost, reweighted, or reorganized during condensation. Versa’s inability to analyze transcripts simultaneously also prohibited counts of code frequency from being preserved throughout the Versa analysis steps. During the analysis comparisons, source awareness may have influenced comparative judgments, particularly for inherently interpretive domains such as thematic robustness and criteria such as narrative depth.

### Conclusion

Using Versa to qualitatively analyze the transcript data still relied heavily on human researchers to come up with prompts and input all the data into the chats. The summaries produced by the Versa analyses were accurate, but lacked depth, and the narrative storytelling that is common in human qualitative analysis was missing. Some of the excerpts Versa selected from the transcripts were misrepresented as direct quotes and failed to include compelling parts of the transcripts. Future research may benefit from using Versa to identify codes and create a thematic analysis outline in collaboration with human researchers thoroughly reviewing the transcripts to develop the bulk of the qualitative analysis and identify meaningful quotes.

## Data Availability

The datasets generated or analyzed during this study are available from the corresponding author on reasonable request.

## Appendix

Multimedia Appendix 1 Interview structure.

Multimedia Appendix 2 Codebook (human-synthesized).

Multimedia Appendix 3 Large language model analysis prompts for Versa Analysis 1.

Multimedia Appendix 4 Large language model analysis prompts for Versa Analysis 2.

Multimedia Appendix 5 Human-synthesized analysis: physical and emotional experiences of fistula.

Multimedia Appendix 6 AI-generated analyses.

## Abbreviations

COM-B: Capability, Opportunity, Motivation–Behavior
HIPAA: Health Insurance Portability and Accountability Act
IDI: in-depth interview
LLM: large language model
LMIC: low- and middle-income country
UCSF: University of California, San Francisco

## References

[1] Hitch D (2024). Artificial intelligence augmented qualitative analysis: the way of the future?. Qual Health Res.

[2] Bijker R, Merkouris SS, Dowling NA, Rodda SN (2024). ChatGPT for automated qualitative research: content analysis. J Med Internet Res.

[3] Li KD, Fernandez AM, Schwartz R, Rios N, Carlisle MN, Amend GM, Patel HV, Breyer BN (2024). Comparing GPT-4 and human researchers in health care data analysis: qualitative description study. J Med Internet Res.

[4] Marshall DT, Naff DB (2024). The ethics of using artificial intelligence in qualitative research. J Empir Res Hum Res Ethics.

[5] Prescott MR, Yeager S, Ham L, Rivera Saldana CD, Serrano V, Narez J, Paltin D, Delgado J, Moore DJ, Montoya J (2024). Comparing the efficacy and efficiency of human and generative AI: qualitative thematic analyses. JMIR AI.

[6] Towler L, Bondaronek P, Papakonstantinou T, Amlôt R, Chadborn T, Ainsworth B, Yardley L (2023). Applying machine-learning to rapidly analyze large qualitative text datasets to inform the COVID-19 pandemic response: comparing human and machine-assisted topic analysis techniques. Front Public Health.

[7] Lennon RP, Fraleigh R, Van Scoy LJ, Keshaviah A, Hu XC, Snyder BL, Miller EL, Calo WA, Zgierska AE, Griffin C (2021). Developing and testing an automated qualitative assistant (AQUA) to support qualitative analysis. Fam Med Community Health.

[8] Tuna M, Schaaff K, Schlippe T (2024). Effects of language- and culture-specific prompting on ChatGPT.

[9] Masoud R, Liu Z, Ferianc M, Treleaven P, Rodrigues M (2023). Cultural alignment in large language models: an explanatory analysis based on Hofstede's cultural dimensions.

[10] Wang W, Jiao W, Huang J, Dai R, Huang J, Tu Z, Lyu M (2024). Not all countries celebrate thanksgiving: on the cultural dominance in large language models.

[11] Yuan H, Che Z, Zhang Y, Li S, Yuan X, Huang L, Hu X, Peng K, Luo S (2025). The cultural stereotype and cultural bias of ChatGPT. J Pac Rim Psychol.

[12] Ahmad I, Dudy S, Ramachandranpillai R, Church K (2024). Are generative language models multicultural? A study on Hausa culture and emotions using ChatGPT.

[13] El Ayadi AM, Obore S, Kirya F, Miller S, Korn A, Nalubwama H, Neuhaus J, Getahun M, Eyul P, Twine R, Andrew EVW, Barageine JK (2024). Identifying opportunities for prevention of adverse outcomes following female genital fistula repair: protocol for a mixed-methods study in Uganda. Reprod Health.

[14] Ahmed S, Genadry R, Asiamah B, Liang M, Tripathi V, Anastasi E (2025). Global, regional and national estimates of obstetric fistula prevalence. BMJ Glob Health.

[15] Barageine JK, Nalubwama H, Obore S, Mirembe E, Mubiru D, Jean A, Akori S, Opio S, Keyser L, McKinney J, Korn AP, Ali S, Byamugisha J, El Ayadi AM (2024). Development and pilot test of a multi-component intervention to support women's recovery from female genital fistula. Int Urogynecol J.

[16] Bigley R, Barageine J, Nalubwama H, Neuhaus J, Mitchell A, Miller S, Obore S, Byamugisha J, Korn A, El Ayadi AM (2023). Factors associated with reintegration trajectory following female genital fistula surgery in Uganda. AJOG Glob Rep.

[17] El Ayadi AM, Barageine JK, Miller S, Byamugisha J, Nalubwama H, Obore S, Korn A, Sukumar S, Kakaire O, Mwanje H, Lester F, Turan JM (2020). Women's experiences of fistula-related stigma in Uganda: a conceptual framework to inform stigma-reduction interventions. Cult Health Sex.

[18] El Ayadi AM, Barageine JK, Neilands TB, Ryan N, Nalubwama H, Korn A, Turan JM (2021). Validation of an adapted instrument to measure female genital fistula-related stigma. Glob Public Health.

[19] Castleberry A, Nolen A (2018). Thematic analysis of qualitative research data: is it as easy as it sounds?. Curr Pharm Teach Learn.

[20] Kiger ME, Varpio L (2020). Thematic analysis of qualitative data: AMEE Guide No. 131. Med Teach.

[21] Michie S, van Stralen MM, West R (2011). The behaviour change wheel: a new method for characterising and designing behaviour change interventions. Implement Sci.

[22] McLeroy KR, Bibeau D, Steckler A, Glanz K (1988). An ecological perspective on health promotion programs. Health Educ Q.

[23] Dedoos.

[24] Sevilla-Liu A (2023). The theoretical basis of a functional-descriptive approach to qualitative research in CBS: with a focus on narrative analysis and practice. J Contextual Behav Sci.

[25] UCSF Versa, Assistants, and API. University of California San Francisco.

[26] (2024). Hello GPT-4o. OpenAI.

[27] OpenAI. UCSF Versa. University of California San Francisco.

[28] Uganda. World Bank Group.

[29] Model release note. OpenAI.

