# Supplementary material for: Efficiency vs Depth in AI-Generated and Human-Synthesized Thematic Analyses of Ugandan Women’s Experiences of Obstetric Fistula: Comparative Qualitative Study
Source: J Med Internet Res. 2026 Oct 9;28:e94809. doi: 10.2196/94809 (PMC13695002; doi:10.2196/94809)
Supplement: Supplementary file 1 [file jmir_v28i1e94809_app1.pdf]

Interview structure [1]

Focus	Example Question
Surgical repair	How easy has it been to access information or advice you needed concerning your health after the fistula repair? *Please explain.*
Experience of post-repair incontinence, fistula repair breakdown, or fistula recurrence.	What have you experienced in terms of ongoing or new leakage or fistula following your surgery?
Physical activities	Please tell me about your activities in and around the house, including any household responsibilities that involve physical activity. By this, I mean any activity that requires you to move around, be active, lift or carry/push things- including cleaning, gardening, and carrying water. *Probe about what these are, how strenuous they are, and how often engaged in.*
Pelvic floor strengthening	[if didn't mention they received guidance on pelvic floor strengthening by fistula providers on physical activity:] What guidance did your fistula healthcare team provide on post-repair pelvic floor exercises? *Please tell me more.*
Nutrition and food security	What do you think about the quantity and quality of food that you're getting since your fistula repair?
Social support and relationships	How have your relationships with your family (not intimate partner) changed since your fistula repair?
Post-repair intimacy and sexual activity	Please tell me about any intimacy with your partner following fistula repair. By this I mean physical (sexual and non-sexual) and emotional intimacy.
If became pregnant after fistula repair	Please tell me about your post-repair pregnancy. *Probe about timing in relation to fistula repair, current status (gestational age or when outcome occurred), planned vs. unintended, and how they felt about the pregnancy.*
Emotional status	How would you describe your mental or emotional state since your fistula repair? *Probe, how do you generally feel? Tell me more about these feelings.*
Intimate partner violence	Please tell me how your intimate partner treats you verbally. *How*

	does your intimate partner speak to you? Probe for level of care and respect, and change since fistula repair.
Substance use	Please tell me about whether you consume alcohol? *Probe about frequency, amount, timing.*
Attitude about repeat surgery	In your view, what do you think a woman who experiences fistula symptoms after having a fistula surgery should do about her symptoms? In your own experience, what have you done about the reoccurrence of your fistula symptoms?
Factors influencing access to fistula care after breakdown	What do you think are some of the reasons why women fail to return to fistula care facilities for care when they experience fistula symptoms after having had surgery? *Probe for individual, community and health system factors*
Recommendations	What advice would you give to other women recovering from fistula surgery about what to do to stay healthy and prevent new leakage/fistula?

References

1. El Ayadi AM, Obore S, Kirya F, Miller S, Korn A, Nalubwama H, Neuhaus J, Getahun M, Eyul P, Twine R, Andrew EVW, Barageine JK. Identifying opportunities for prevention of adverse outcomes following female genital fistula repair: protocol for a mixed-methods study in Uganda. Reprod Health 2024 Jan 4;21(1):2. doi: 10.1186/s12978-023-01732-7

This is a Multimedia Appendix to a full manuscript published in the J Med Internet Res. For full copyright and citation information see http://dx.doi.org/10.2196/jmir.94809
