# Supplementary material for: Efficiency vs Depth in AI-Generated and Human-Synthesized Thematic Analyses of Ugandan Women’s Experiences of Obstetric Fistula: Comparative Qualitative Study
Source: J Med Internet Res. 2026 Oct 9;28:e94809. doi: 10.2196/94809 (PMC13695002; doi:10.2196/94809)
Supplement: Supplementary file 2 [file jmir_v28i1e94809_app2.pdf]

Codebook (Human-synthesized)

N	Parent code	n	Child code	Code definition
1	**Surgical experiences**<br><br>Any description of general surgical experiences a woman had during the fistula repair. This includes surgery physical and emotional experiences. This also includes participant narratives focused on the fistula surgical hospitalization period but excludes only counseling which should be coded under health info.	1.1	**Physical experiences related to the surgery**	Any discussion of surgery or post-surgery (within the surgical hospitalization) physical symptoms including pain, leakage, complications, healing, etc.
		1.2	**Emotional experiences related to the surgery***	Any discussion of emotional response/experience to the surgery or post-surgery (within the surgical hospitalization).
		1.3	**Quality of care perceptions**	Any description of fistula patients' perceptions about the quality of care they received at the fistula repair facility. *Double code with appropriate domain codes.*
2	**Social support**<br><br>Any description of emotional, informational, tangible or appraisal support that a woman discusses referring to any time period.	2.1	**Emotional support***	Any discussion of receipt of verbal and nonverbal communication of care and concern (e.g., reassurance, empathy, comfort, and acceptance). *Double code with appropriate domain codes.*
		2.2	**Informational support**	Any discussion of receipt of advice, guidance, information or knowledge to help someone solve problems, make decisions, or

				understand a situation better. *Double code with appropriate domain codes.*
		**2.3**	**Tangible support***	Any discussion of receipt of concrete direct assistance involving physical action, material goods, or financial assistance (e.g., paying for care, giving a gift, cleaning, or watching kids, bringing water). *Double code with appropriate domain codes.*
		**2.4**	**Appraisal support***	Any discussion of receipt of information that helps them evaluate their abilities and experiences, often in the context of a stressful or challenging situation. *Double code with appropriate domain codes.*
**3**	**Coping strategies**			Any description of coping: actions, thoughts, and behaviors used to manage stressful situations including problem-focused, emotion-focused, or social support-seeking strategies. This includes modifying activities to comply with post-surgical guidelines and behaviors to address leakage. *Double code with appropriate domain codes.*
**4**	**Health information access**<br><br>Any description of	**4.1**	**Counseling – at surgical hospitalization**	Any description of the counselling the fistula patients received while at the fistula repair facility both pre and post repair to help in their

	what, how and from whom fistula patients received information concerning their fistula repair after discharge from hospital. This also includes ease of access to information needed. *Double code with appropriate domain codes.*			recovery regardless. This includes guidance on nutrition, physical activity, sexual resumption, fertility, post-repair pregnancy and delivery mode, etc. Code for both content, experience, and any perspectives on quality of counseling received. *Double code with appropriate domain codes*<br><br>*Where unmet counseling needs were expressed, please double code with the counseling timing code (4.1 or 4.2), the unmet need code (15), and the appropriate domain code (8).*
		**4.2**	**Counseling – after hospital discharge**	Any description of the counselling the fistula patients received after fistula surgery-related hospital discharge (when they are at home/in their community) to help in their recovery. This includes guidance on nutrition, physical activity, sexual resumption, fertility, post-repair pregnancy and delivery mode, etc. Code for both content, experience, and any perspectives on quality of counseling received. *Double code with appropriate domain codes.*<br><br>*Where unmet counseling needs were expressed, please double code with the counseling timing*

				code (4.1 or 4.2), the unmet need code (15), and the appropriate domain code (8).
**5**	**Source of support or info**	**5.1**	**Providers/healthcare workers**	Any discussion of **<u>who is providing</u>** the informational, emotional, tangible, or appraisal support OR health information. This may include (but is not limited to) providers, family, peers, community members (e.g., neighbors, religious communities, other groups), and any others.
		**5.2**	**Peers/community member**	
		**5.3**	**Relatives/family members**	
		**5.4**	**Partner**	
		**5.5**	**Other***	
**6**	**Stigma**		Any discussion of enacted, internalized, or anticipated stigma. *Double code with appropriate source or domain codes.*	
**7**	**Impact of counseling and implementation**	**7.1**	**Impact on healing***	Any discussion around the possible impact of domain-specific counseling on fistula outcomes including both positive (e.g., recovery) and negative (e.g., recurrence/breakdown, pain, incontinence, etc). *Double code with appropriate domain codes.*
		**7.2**	**Specific guidance on domain**	Any description of the guidance provided on domain-specific or limitations. *Double code with appropriate domain codes.*
		**7.3**	**Post surgery guidelines on implementing the domain**	Any description of how the fistula patients managed to follow the post-surgical repair guidelines they were given by health workers. *Double code with appropriate domain codes*.

		7.4	**Barriers to implementing**	Any description of how the fistula patients managed to follow the post-surgical repair guidelines and any barriers to implementation. *Double code with appropriate domain codes*.
		7.5	**Facilitators to implementing**	Any description of how the fistula patients managed to follow the post-surgical repair guidelines and any facilitators/help towards implementation. *Double code with appropriate domain codes*.
**8**	**Domains/topic areas for counseling**<br><br>*To be double coded with 7 above on the domains.*	**8.1**	**Physical function and activities**	Any discussion of post repair exercise, movement, work, lifting etc. and daily living and exercise.
		**8.2**	**Pelvic floor health and exercises**	Any discussion of post-repair pelvic floor exercises experiences by fistula patients, demonstrating their understanding and practice of these exercises.
		**8.3**	**Nutrition and food security**	Any discussion around the post-repair nutrition and food security experiences by fistula patients. This includes challenges, facilitator and guidance given. Also includes beverages including caffeine, diuretics (and any counseling to reduce these- tea, coffee, energy drinks).
		**8.4**	**Social and relational dynamics**	Any discussion around the post-repair relationship experiences by fistula patients including any changes in relationship type or

				quality.
		**8.5**	**Intimacy and sexuality**	Any discussion around resuming sex and Pregnancy prevention after fistula repair, inclusive of interests, fears, beliefs, contraceptive use, the guidance given with its implementation challenges and facilitators.
		**8.6**	**Post repair – pregnancy**	Any discussion around post repair pregnancy intention, preferences, family planning, etc.
		**8.7**	**Other topics***	Any other counseling topic.
**9**	**Emotional and mental well being**	**9.1**	**Post repair – emotions**	Any discussion around post repair emotional experiences inclusive of emotional changes noted plus the associated challenges and facilitators. *Double code with appropriate coping strategy or other codes.*
**10**	**Intimate partner violence (IPV)***	**10.1**	**Post repair – IPV***	Any discussion around post repair intimate partner violence experiences (threats, anticipations, or actions) after fistula repair, inclusive of physical, sexual, emotional, or psychological harm inflicted by a current or former intimate partner or spouse. It encompasses a wide range of abusive behaviors, including physical violence, sexual coercion, psychological abuse, and controlling behaviors. It may reflect

				on its possible impact on fistula repair breakdown, any IPV disclosure and support sought.
		**10.2**	**Post repair – IPV impact***	Any discussion around the possible impact of post-repair intimate partner violence experiences on post repair fistula outcomes including both positive (e.g., recovery) and negative (e.g., recurrence/breakdown, pain, incontinence, etc.). *Double code with appropriate domain codes.*
		**10.3**	**Post repair – IPV support***	Any discussion of support or interventions for IPV (whether sought or not).
**11**	**Substance use***	***11.1***	**Post repair – substance use***	Any discussion around post repair substance (drug and alcohol) use experiences after fistula repair, and changes in substance use after repair plus its possible impact on fistula repair breakdown etc.
		**11.2**	**Post repair – substance use guidance***	Any discussion around post repair substance guidance given.
**12**	**Repeat surgery and associated care***	**12.1**	**Attitudes towards surgery***	Any discussion of views towards repeat surgery both negative and positive, including perceived chances of success.
		**12.2**	**Barriers towards surgery***	Any discussion of perceived or actual barriers to undergoing subsequent fistula repair surgery (could be individual level barriers or systemic/health care system or

				provider related).
		12.3	**Repeat surgery care seeking**	Any description of efforts made by the woman with fistula to seek care when fistula symptoms recurred. *Double code with other relevant codes.*
13	**Other (non-surgical) care**		**Other care**	Any description of other medical (e.g., physicians, nurses, pharmacists) or traditional health care seeking (e.g., herbalists) or receipt. *Double code with appropriate domain codes.*
14	**Post repair symptoms and experiences**		Any description of experiences of fistula symptoms like incontinence, breakdowns after surgery. Inclusive of perceived causes, emotions felt, actions taken and coping mechanisms. *Double code with appropriate domain codes.*	
15	**Unmet needs**		Any description of specific needs that the study participant brings up. This is likely to be across social support domains and others. *Double code as appropriate.*	
16	**Disclosure**		Any discussion of disclosure of fistula or fistula history. *Double code with stigma, needs, other domain codes.*	
17	**Recommendations***		Any suggestions mentioned around what should be done to prevent fistula recurrence/breakdown including advice on the role of family/community and healthcare providers in prevention of fistula adverse outcomes after repair.	
18	**Sociocultural norms including gender***		Any discussion of cultural practices by household or community including reference to customs, social pressures, ceremony, or community beliefs, including not directly related to health. Include references to gender, which encompasses beliefs and attitudes related to the characteristics and roles of women or men, or differences between men and women. These include personal beliefs and attitudes as well as perceptions of community or	

			societal norms related to gender, i.e. how men or women are expected to behave. Includes discussions about people who may be violating or upholding/reinforcing social or gender norms.
**19**	**Socioeconomic status**		Any description of the respondent (or household) income, occupation, and economic standing/class, including inequities in access to resources, plus issues related to privilege, power and control. Includes discussions of poverty, etc.

*Codes with no LLM-generated equivalent

This is a Multimedia Appendix to a full manuscript published in the J Med Internet Res. For full copyright and citation information see http://dx.doi.org/10.2196/jmir.94809
