# Supplementary material for: Efficiency vs Depth in AI-Generated and Human-Synthesized Thematic Analyses of Ugandan Women’s Experiences of Obstetric Fistula: Comparative Qualitative Study
Source: J Med Internet Res. 2026 Oct 9;28:e94809. doi: 10.2196/94809 (PMC13695002; doi:10.2196/94809)
Supplement: Supplementary file 4 [file jmir_v28i1e94809_app4.pdf]

Large language model analysis prompts for Versa Analysis 2

Versa Analysis 2 (deductive). Prompts for the analyses of all 17 transcripts.

***Me [Prompt 5]**: I am going to give you interview transcripts, and I want you to qualitatively analyze them using the uploaded codebook. Identify themes and provide excerpts from the transcript as examples.*
The research question is: What are Ugandan women's experiences of obstetric fistula, fistula repair surgery, and recovery?
Refer to each transcript by the Study ID.

***Versa**: [output]*

***Me**: Transcript for study ID [number]*
[insert transcript]

***Versa [Output 5]**: [output]*

***Me [Prompt 6]**: Now, create an overall analysis of the transcript. The research question is: What are Ugandan women's experiences of obstetric fistula, fistula repair surgery, and recovery?*
During analysis, for "What are Ugandan women's physical and emotional experiences of fistula?" consider "What physical symptoms of fistula did they experience?" and "What were their emotional experiences about having fistula?"
During analysis, for "What are Ugandan women's physical and emotional experiences related to fistula repair surgery?" consider physical and emotional experiences related to the surgery.
During analysis, for "What are Ugandan women's experiences of recovery post-repair?" focus on recovery experiences, not specific guidance. These experiences should take place within the first few months after surgery, when the guidance was applicable. Ex: during the recovery period the participant returned to work digging because she had to ensure income, or participant was able to rest during recovery because her children fetched water for her. Consider "What experiences hindered their recovery?" and "What experiences assisted their recovery?"

***Versa [Output 6]**: [output]*

Versa Analysis 2 (deductive). Prompt to combine Versa outputs into an overall analysis.

***Me [Prompt 7]**: I am going to give you an overall qualitative analysis for 17 different study IDs. I want you to condense all of the 17 analyses into one final analysis.*

***Versa**: [output]*

***Me**: [insert all 17 Output 6s]*

***Versa**: [output]*

***Me**: include excerpts*

***Versa [Output 7]**: [output]*

[1]

For the purpose of this study, only the Versa output in response to the prompt of Ugandan women's experiences of obstetric fistula was analyzed. The Versa output in response to the prompt of Ugandan women's experiences of fistula repair surgery and recovery was excluded.

References

1. OpenAI. UCSF Versa.

This is a Multimedia Appendix to a full manuscript published in the J Med Internet Res. For full copyright and citation information see http://dx.doi.org/10.2196/jmir.94809
