# Supplementary material for: Efficiency vs Depth in AI-Generated and Human-Synthesized Thematic Analyses of Ugandan Women’s Experiences of Obstetric Fistula: Comparative Qualitative Study
Source: J Med Internet Res. 2026 Oct 9;28:e94809. doi: 10.2196/94809 (PMC13695002; doi:10.2196/94809)
Supplement: Supplementary file 5 [file jmir_v28i1e94809_app5.pdf]

Human-synthesized analysis: Physical and emotional experiences of fistula

Women reported a variety of physical and emotional experiences of fistula, both before surgery and after the initial three-month recovery period post-repair. Experiences related to the surgery and immediate three-month recovery period were not included in this analysis. Descriptions of urinary incontinence due to the fistula, its management, and the specific impact it had on women's daily lives were common physical experiences reported. The emotional experiences were overwhelmingly negative and suggested a notable impact of fistula on women's mental health.

Physical: Urinary Incontinence While Sleeping

Lying down to sleep at night increased urinary incontinence in several women, even in women who could feel the urge to urinate and pass it on their own during the day. A 39-year-old woman said, *"I don't get urine incontinence when I am standing but I feel urine flowing on my thighs when I am sleeping."* Women struggled with their bedding becoming soaked in urine while they slept. A 35-year-old woman reported, *"I was leaking heavily that even when I would sleep, urine would flow up to the head and my hair would get soaked in urine. I started developing sores all over my body because of the urine."*

Physical: Wearing a Diaper or Padding

Management of urinary incontinence involved the use of diapers, cloths, or polyethylene paper. Women described padding themselves with these materials to manage their urine leakage, most often when leaving home to go somewhere. For example, women described padding in circumstances such as taxi rides where there is no immediate access to a bathroom or if a woman is working with clients or customers and cannot take breaks to pass urine. Some women explained they prefer using diapers for incontinence management because the urine-soaked cloths or polyethylene paper burn their skin and cause sores.

Emotional: Negative Emotions

Fistula can be very demoralizing and upsetting to women struggling with it. Feeling hopeless, sad, upset, and stressed were very common emotional experiences reported among the women due to the physical implications of fistula. In particular, women who experienced a fistula repair

breakdown and persistence incontinence reported feeling hopeless that a full recovery would be possible. A 34-year-old woman said, *“[When I got a leakage] I felt so bad and even now, I am unable to sleep because I am always thinking about the leakage. I am uncomfortable and sad.”* Similarly, a 49-year-old woman explained, *“I am not the same anymore; I lost my happiness when I started leaking urine.”* One woman shared that she had suicidal thoughts because she could not fully heal from her fistula.

A 44-year-old woman said that there are times she doesn’t go to work if she doesn’t have diapers due to a lack of money, because when she uses cloths to pad herself, the odor is too strong even immediately after washing. She described the emotional toll of needing padding to cope with fistula urine incontinence: *“There’s no [feeling] of happiness at all… my heart is not at peace. I am not happy with myself. I keep asking myself why the urine keeps leaking. Sometimes you go to dry the cloths on the hanging line. People keep seeing one soiled towel and cloth. Every single time. When new neighbors come, you get worried. When you see people who know you that you keep drying the same thing. When you have no soap, you just dry them without washing. Whoever passes smells them. So, you feel your heart is tired."*

<u>Emotional: Self-hatred</u>

Women’s self-perspective was significantly impacted by their fistula. Multiple women reported hating themselves because of the fistula and its impact. Women’s emotional responses to fistula were complicated by their perceptions of why they had experienced fistula; for example, several women shared that they believed it was their fault that they got a fistula and struggled with urinary incontinence, particularly when they were significantly slower to heal than other women, which in some cases was caused or exacerbated by experiencing stigmatization by members of their community. The common experiences of both enacted and internalized stigma played a role in the emotional experience. For example, one 20-year-old woman said, *“In my heart, I feel I have hated myself because of this condition.”* A different 20-year-old participant shared that when she started leaking again after having undergone fistula repair, she became depressed: she was crying, not eating, not leaving her bed, and hated herself. Similarly, a 42-year-old woman explained that when she got a fistula, she hated herself and would isolate herself because she felt so bad. The emotional experiences of women with fistula pertaining to their fistula were overwhelmingly negative.

This is a Multimedia Appendix to a full manuscript published in the J Med Internet Res. For full copyright and citation information see http://dx.doi.org/10.2196/jmir.94809
