# Supplementary material for: Efficiency vs Depth in AI-Generated and Human-Synthesized Thematic Analyses of Ugandan Women’s Experiences of Obstetric Fistula: Comparative Qualitative Study
Source: J Med Internet Res. 2026 Oct 9;28:e94809. doi: 10.2196/94809 (PMC13695002; doi:10.2196/94809)
Supplement: Supplementary file 6 [file jmir_v28i1e94809_app6.pdf]

AI-generated analyses

Versa Analysis 1 (inductive): Physical and emotional experiences of fistula

The following section is directly from Versa's output, except for the participant labels after the quotes which were relabeled to mask the study IDs.[1]

Physical Symptoms:

All participants reported severe physical symptoms, predominantly characterized by continuous urine leakage, which significantly impaired their daily activities and physical health. The leakage often persisted despite multiple surgeries, leading to discomfort, pain, and the need for frequent changing of pads or makeshift solutions. Additional complications included back pain, kidney issues, and physical sores due to prolonged exposure to urine.

Excerpts:

- *"The physical symptoms experienced by the respondent include continuous leakage of urine, which started two days after delivering a stillbirth." (Participant 4)*
- *"The participant experienced continuous leakage of urine immediately after the removal of the catheter post-caesarean section." (Participant 11)*
- *"I left hospital when I was still leaking. Every time I am operated on, I leave the hospital when I am still leaking." (Participant 8)*
- *"Urine started leaking again 2 days after the surgery before the catheter was removed." (Participant 3)*

Emotional Impact:

Emotionally, living with fistula led to profound distress, embarrassment, and social stigma. The constant leakage and associated smell caused feelings of isolation, self-hatred, and hopelessness. Many women felt stigmatized and abandoned by their communities and families, exacerbating their emotional burden. The emotional toll included depression, suicidal thoughts, and a sense of despair, compounded by the social implications of their condition.

Excerpts:

- *“Emotionally, the respondent faced significant distress and embarrassment due to the fistula.” (Participant 4)*
- *“The emotional impact of fistula was profound, with feelings of embarrassment, helplessness, and sadness.” (Participant 11)*
- *“They think it may be infectious. Yeah, I see that it has been a big challenge with the family. They don’t get close to me that much.” (Participant 8)*
- *“In my heart, I feel I have hated myself because of this condition.” (Participant 16)*

Versa Analysis 2 (deductive): Physical and emotional experiences of fistula

The following section is directly from Versa’s output, except for the participant labels after the quotes which were relabeled to mask the study IDs.[1]

Physical Symptoms and Impact

Obstetric fistula caused debilitating physical symptoms, primarily persistent, uncontrollable urinary leakage. Women reported how urine leakage disrupted their daily lives, causing discomfort, skin irritation, and hygiene challenges. Many relied on improvised materials such as plastic bags, cloth, or baby diapers to manage leakage.

Excerpts:

- *“I spent the night soaked in urine. I was in a pool of urine on the bed. I was like that with my child on the bed.”* (Participant 15)
- *“The urine leaks when I have taken a lot of water. I use the baby pampers. At night when I am sleeping, it flows nonstop.”* (Participant 17)
- *“Before, I was leaking heavily that even when I would sleep, urine would flow up to the head and my hair would get soaked in urine.”* (Participant 7)

Physical symptoms were often compounded by additional health issues, including back pain, kidney problems, labor-like contractions, and difficulty passing stool.

Excerpts:

- *“At first, I had some cramps that were similar to labor-like contractions because it felt like I had to push a baby out.”* (Participant 5)
- *“During one of the previous surgeries, I don’t know if they reduced my passage for faeces. I now have difficulty in passing stool.”* (Participant 9)

<u>Emotional Distress</u>

The emotional toll of fistula was profound. Women described feelings of shame, despair, isolation, and loneliness. Many were stigmatized and ridiculed by their communities, facing rejection at work, in social settings, and even within their families. Spousal relationships often deteriorated due to the condition, leading to separation or abandonment.

<u>Excerpts:</u>

- *“People said a lot of things about me and I got fed up. The customers tell me that I am smelling.”* (Participant 1)
- *“When new neighbors come, you get worried. When you see people who know you…Whoever passes smells them. So, you feel your heart is tired.”* (Participant 8)
- *“My husband can insult me that I am leaking. He says, ‘Keep your urine to yourself!’”* (Participant 2)
- *“Some people used to come and tell me that you won’t heal. They can’t operate a bladder. It is just a polyethene bag. Can you pass a needle through a polyethene bag? You are meant to die.”* (Participant 7)

<u>Coping Mechanisms</u>

Despite immense challenges, women demonstrated remarkable resilience. Faith in God and religious practices emerged as key coping mechanisms, providing hope and emotional strength.

<u>Excerpts:</u>

- *“I cry and later wipe my tears away. Other times I cry to God saying, ‘What did I ever do to you?’”* (Participant 1)

- *“I became a born-again Christian after getting the labor-like contractions because I wanted God to heal me, and he did. However, the fistula didn’t heal.”* (Participant 5)

Some women found solace in connecting with other fistula survivors, forming bonds of mutual understanding and support.

References

1. OpenAI. UCSF Versa.

This is a Multimedia Appendix to a full manuscript published in the J Med Internet Res. For full copyright and citation information see http://dx.doi.org/10.2196/jmir.94809
